# A standardized online clinical education and assessment tool for neurology clerkship students assigned to multiple sites

**DOI:** 10.1007/s40037-013-0097-5

**Published:** 2013-12-05

**Authors:** Neil R. Holland, Ilya Grinberg, David Tabby

**Affiliations:** 1 The Neuroscience Institute, Monmouth Medical Center, 107 Monmouth Rd, West Long Branch, NJ 07764 USA; 2 Department of Neurology, Drexel University College of Medicine, Philadelphia, PA USA

**Keywords:** Medical education, Neurology clerkship, Online education, Online assessment

## Abstract

The Drexel neurology clerkship experience can vary from large groups at a university hospital inpatient unit to smaller groups at private physician offices. Evaluations are based on the site director’s subjective assessment and performance on the National Board of Medical Examiners neurology shelf examination. We are developing a standardized online clinical neurology course and quiz for the whole clerkship. We piloted the course and quiz at a single site for one academic year and compared their test scores with a control group of students at other clerkship sites who took the online quiz without viewing the course. Students at the pilot site achieved higher scores both on the neurology shelf examination and the clinical quiz and also reported higher teaching satisfaction scores than students at all other sites. There was a 97 % participation rate in the online quiz from the other sites. Use of this online course and quiz provides effective standardized clinical neurology teaching and evaluation that can be applied to students across multiple sites.

**Electronic supplementary material:**

The online version of this article (doi:10.1007/s40037-013-0097-5) contains supplementary material, which is available to authorized users.

The Drexel University College of Medicine neurology clerkship is a required 4-week rotation in the 4th year curriculum taken at a variety of different sites, some in large group rotations of 12–15 students assigned to Hahnemann University Hospital in Philadelphia, others in groups of 1–4 students assigned to smaller community hospitals and private physician offices at affiliated campuses across Pennsylvania and New Jersey. Students are assigned to their clerkship sites using a lottery system. Student feedback indicated that those assigned to small-group rotations with private practitioners were less satisfied with the didactic teaching they received than students assigned to rotations at the university hospital. However, private practitioners may find it difficult to take time out of their busy clinical schedules for formal teaching [1]. A 2005 survey of neurology clerkship directors from the American Academy of Neurology (AAN) showed that more than one-third of neurology clerkship students are assigned to private physicians’ offices or to inpatient units at community hospitals for their rotations [2]. The lack of opportunities for didactic teaching with students’ rotation sites spread so thinly is therefore not unique to our institution.

There would appear to be a need for a standardized clinical neurology course that can be accessed by clerkship students at multiple sites; online medical education tools have been effective in this role [3–5] and those developed specifically for neurology have included video clips demonstrating individual physical signs [6], a neurology e-textbook [7], and an online course aimed at postgraduate medical trainees [8].

Our students are graded based on a composite of a subjective review by one of many different site directors and their performance on the National Board of Medical Examiners neurology shelf examination. This approach to student grading has been criticized for being too subjective or too reliant on shelf examination scores [9, 10]. Many students who fail the shelf examinations do well on clinical ratings and later match for residency, suggesting that these examinations do not predict later clinical performance but simply measure reading and test taking skills [11]. Furthermore, the validity of standard medical student assessment forms is dependent on the number or raters [12], and our neurology students assigned to smaller groups usually work with a single clinician preceptor. A proctored clinical examination with real or standardized patients is a better predictor of student competence [10, 13], but this approach is time consuming, expensive, and difficult when students are rotating at multiple sites.

We are developing an online integrated symptom-based clinical neurology course with an associated quiz to add standardized didactic clinical teaching and assessment to the existing clerkship experience for all of our students across multiple clinical sites. The course consists of neurology lectures covering: Neurological localization of muscle weakness, Abnormal movements, Abnormal gait, Dizziness, The unconscious patient, Headache and facial pain, Change in mental status, Abnormal speech, and Fits and faints. These topics were selected based on the AAN clerkship core curriculum guidelines [14]. Lectures are organized by presenting symptoms, not by individual physical signs or diseases as they are in currently available online neurology education tools. Each lecture emphasizes the physical diagnosis using clinical images and video clips from patients, with consent and/or masking to make them unidentifiable, and from other published educational sources. The course ends with a neurology quiz consisting of clinical images or video clips each followed by 1–3 questions. There are 1–2 questions taken from every lecture, selected to emphasize the key clinical concepts presented (supplemental online table). Only two of the video clips featured in the quiz were taken from the course. One of them is a clip of a patient with chorea, which was labelled as drug-induced dyskinesias in the course, but in the quiz was shown with clinical information suggesting a diagnosis of Huntington’s chorea. None of the other clinical material included in the quiz had been seen before by any of the students. The quiz is followed by questions soliciting student satisfaction with teaching, preparedness for the quiz, and difficulty of the quiz. These are graded on a scale of 1 through 5, where 5 is most satisfied. A video segment illustrating some of the course content and quiz can be viewed as supplemental material online.

We piloted the course at a neurology private practice site that accepts one or two students from our institution for their neurology clerkship assignments each block. All ten students assigned to that pilot site during the 2012–2013 academic year completed the neurology online course and took the quiz during their clerkship. The control group consisted of 27 students taking their clerkship at all other sites during one block that same academic year. Fourteen of the 27 control students were assigned to one large group at the university hospital, and 13 were assigned to smaller affiliated community hospitals and practices in groups of 1–4. They were each emailed a link and time-limited access code for the quiz, which they completed online while they were at their respective clerkship sites, without first viewing the video course. This study was approved by the university’s institutional review board.

Students at the pilot site achieved higher scores on both the neurology shelf examination and neurology clinical quiz than the control group. The effect on neurology shelf scores was more significant when compared with students at other small sites than students assigned to the University Hospital. Students at the pilot site reported higher satisfaction scores for didactic teaching, and although they felt better prepared for the quiz than students at other sites, all students rated the quiz as equally difficult (Table 1). The rates of correct identification of Huntington’s chorea, which the pilot group students had seen before with an alternate diagnosis, were the same in all groups, so students at the pilot site were not simply learning to recognize the video clips. During the block when all students took the online clinical quiz, the participation rate was 97 %, even though students were located at eight different sites at the time.

**Table 1:** Examination and satisfaction scores from students at the pilot site versus the control groups, [mean (SD)]

	Pilot site (*n* = 10)	Controls		
		Total (*n* = 27)	Small sites (*n* = 13)	University (*n* = 14)
Neurology shelf examination raw score (0–100)	76 (6)	72 (5.8)*	71 (5)*	73 (6)^a^
Clinical quiz (0–42)	32.7*(3.4)	28.4 (3.8)*	28.1 (4.0)*	28.6 (3.6)*
Satisfaction with teaching (1–5)	4.5	3.3*	3.2*	3.4*
Preparedness for clinical quiz (1–5)	4.5	3.3*	3.5*	3.1*
Difficulty of clinical quiz (1–5)	3.1	3.9^a^	3.9^a^	3.9^a^

* *P* < 0.05, 2-tailed *t* test

^a^Not statistically significant

These data indicate that an online integrated symptom-based clinical neurology course improved both student perception of teaching and performance on the standardized neurology shelf examination, particularly when comparing students assigned to other small sites. Although it was not surprising that students at the pilot site performed better on the neurology clinical quiz, since the quiz was based on the course, these data do show that the online course was an effective teaching tool. Finally, the clinical quiz was hosted online and was used at little or no cost to assess clinical neurology knowledge for students assigned to multiple clerkship sites. We were able to achieve near-total participation by simply emailing each student a link to the online site.

We recognize that these conclusions are limited by small numbers. In addition, the students at the pilot site might have received better teaching overall, independent of the online course, which could have affected their satisfaction and test scores. The course is designed to supplement not replace the conventional clerkship experience. The content tested in the clinical quiz is limited to 1–2 key learning points from each lecture, and is not all-inclusive. Finally, the clinical quiz does not replace our grading system, but does allow us to more easily add an objective clinical measure to the current composite grade. We plan to use a modified version of this course and quiz to standardize didactic teaching and clinical assessment for our neurology students across all our clerkship sites, and we will continue to collect further data to validate these pilot site results and refine our neurology clerkship core curriculum. We believe that this type of teaching and assessment tool could ultimately be applicable to other large medical institutions with multi-site neurology clerkships.

## Electronic supplementary material

Below is the link to the electronic supplementary material.

Supplementary material 1 (MP4 12482 kb)

Supplementary material 2 (DOCX 14 kb)
