# Supplementary material for: A standardized online clinical education and assessment tool for neurology clerkship students assigned to multiple sites
Source: Perspect Med Educ. 2013 Dec 5;3(1):41–5. doi: 10.1007/s40037-013-0097-5 (PMC3889993; doi:10.1007/s40037-013-0097-5)
Supplement: Supplementary file 2 — Supplementary material 2 (DOCX 14 kb) [file 40037_2013_97_MOESM2_ESM.docx]

**Supplemental Table. Neurology knowledge emphasized in the clinical quiz:**

| Recognize spasticity and know common CNS causes. |
| --- |
| Recognize fasciculations from motor neuron disease. |
| Recognize and localize Broca’s aphasia. |
| Recognize and lateralize peripheral nystagmus. |
| Recognize subarachnoid hemorrhage on a CT scan, and know the next diagnostic and management steps. |
| Recognize epidural hematoma and herniation on a CT scan, and identify ipsilateral weakness as a false localizing sign from Kernohan’s notch. |
| Recognize an absence seizure.  Distinguish primary from secondarily generalized seizures including brain imaging, age of onset and EEG findings. |
| Distinguish delirium from dementia. |
| Distinguish locked-in syndrome from coma and vegetative state. |
| Recognize proximal weakness from myopathy. |
| Diagnose Huntington’s disease based on clinical video of chorea and family history. |
| Recognize and localize an ulnar claw hand. |
| Diagnose S1 root lesion based on distribution of sciatica pain and absent ankle jerk. |
| Recognize early Parkinson’s disease. |
| Identify supranuclear gaze palsy and diagnose PSP. |
| Identify an ataxic gait. |
| Recognize Horner’s syndrome and with the clinical history provided diagnose carotid dissection. |
| Recognize conversion disorder. |
| Recognize familial essential tremor. |
